# Preemptive interferon-α treatment could protect against relapse and improve long-term survival of ALL patients after allo-HSCT

**DOI:** 10.1038/s41598-020-77186-9

**Published:** 2020-11-19

**Authors:** Sining Liu, Xueyi Luo, Xiaohui Zhang, Lanping Xu, Yu Wang, Chenhua Yan, Huan Chen, Yuhong Chen, Wei Han, Fengrong Wang, Jingzhi Wang, Kaiyan Liu, Xiaojun Huang, Xiaodong Mo

**Affiliations:** 1 Peking University People’s Hospital, Peking University Institute of Hematology, National Clinical Research Center for Hematologic Disease, Research Unit of Key Technique for Diagnosis and Treatments of Hematologic Malignancies, Chinese Academy of Medical Sciences, 2019RU029, Beijing Key Laboratory of Hematopoietic Stem Cell Transplantation, No. 11 Xizhimen South Street, Xicheng District, Beijing, 100044 China; 2 Peking-Tsinghua Center for Life Sciences, Academy for Advanced Interdisciplinary Studies, Peking University, Beijing, China

**Keywords:** Leukaemia, Acute lymphocytic leukaemia

## Abstract

Relapse was the major cause of treatment failure in patients with acute lymphoblastic leukemia (ALL) after allogeneic hematopoietic stem cell transplantation (allo-HSCT). We aimed to identify the efficacy and safety of preemptive interferon-α (IFN-α) treatment in ALL patients who had minimal residual disease (MRD) after allo-HSCT. Multiparameter flow cytometry and polymerase chain reaction assays were applied for MRD monitoring. Recombinant human IFN-α-2b injections were administered subcutaneously twice weekly in every 4 weeks cycle. Twenty-four (35.3%), 5 (7.4%), 6 (8.8%), and 13 (19.1%) patients achieved MRD negativity at 1, 2, 3, and > 3 months, respectively, after treatment. Seven patients showed grade ≥ 3 toxicities after IFN-α treatment. The 4-year cumulative incidence of total acute graft-versus-host disease (aGVHD), severe aGVHD, total chronic GVHD (cGVHD), and severe cGVHD after treatment was 14.7%, 2.9%, 40.0%, and 7.5%, respectively. The 4-year cumulative incidences of relapse and non-relapse mortality after treatment was 31.9% and 6.0%, respectively. The 4-year probabilities of disease-free survival and overall survival after IFN-α treatment were 62.1% and 71.1%, respectively. Thus, preemptive IFN-α treatment could protect against relapse and improve long-term survival for ALL patients who had MRD after allo-HSCT. The study was registered at https://clinicaltrials.gov as #NCT02185261 (09/07/2014).

## Introduction

Despite considerable advances in the allogeneic hematopoietic stem cell transplantation (allo-HSCT)^1^, relapse remains the major cause of transplant failure in patients with acute lymphoblastic leukemia (ALL)^2^. Thus, identifying patients who were at higher risks for relapse after allo-HSCT is of great importance. Minimal residual disease (MRD) helped to identify patients who still harbored higher levels of disease but were below the detection capabilities of morphological analysis. Multiparameter flow cytometry (MFC) identified cells with leukemia-associated immunophenotypes (LAIPs) and polymerase chain reaction (PCR) assays detected leukemia-associated genetic abnormalities, both could be applied for monitoring MRD in leukemia patients. MRD monitoring was proved to predict impending relapse after allo-HSCT by numerous studies^3–5^.

Impending relapse could be prevented by the early detection of MRD and timely treatments. Thus, preemptive intervention, which could spare patients in remission from further therapies, was reasonable for patients with MRD. Chemotherapy plus donor leukocyte infusion (Chemo-DLI) was the most effective preemptive intervention for MRD^6,7^, however, it may lead to some severe complications (e.g., graft-versus-host disease [GVHD] and pancytopenia)^8^. In addition, it was out of choices for some patients because of related donor refusal or unavailability of the second donation from an unrelated donor. Preemptive tyrosine kinase inhibitor (TKIs) treatment was proved to be a useful intervention^9,10^, but only applied to patients with Philadelphia chromosome (Ph)-positive ALL. Chimeric antigen receptor (CAR) T-cell immunotherapy was another potential preemptive intervention^11–13^. However, it might also lead to several complications (e.g. life-threatening neurological toxicity and cytokine release syndrome)^14,15^, and remissions after CAR T-cell treatment was relatively brief because of poor CAR T cell persistence and/or leukemia cell resistance^16^.

Interferon-α (IFN-α) had shown activity in acute leukemia through immune activation^17^, which rekindled the interest of using IFN-α as an immunotherapy for patients receiving allo-HSCT^18^. Our pilot studies showed that IFN-α was a safe agent for allo-HSCT recipients^19^. We further confirmed that preemptive IFN-α treatment can clear the MRD effectively in patients with acute leukemia and high-risk myelodysplastic syndrome after allo-HSCT^4,20–22^. IFN-α could also be used as a salvage treatment for patients with unsatisfactory response to preemptive Chemo-DLI^23^. However, the sample of ALL patients enrolled in these studies was relatively small, and no study had identified the efficacy of preemptive IFN-α treatment in a disease-specific population of patients with ALL after allo-HSCT. In addition, the follow-ups of these patients were short. Thus, the long-term efficacy of preemptive IFN-α treatment remains unknown in ALL patients following allo-HSCT.

Therefore, we aimed to identify the safety and long-term efficacy of preemptive IFN-α treatment in ALL patients following allo-HSCT.

## Results

### Patient characteristic

The characteristics of the 68 ALL patients receiving preemptive IFN-α treatment are summarized in Table 1. Besides of *WT1*, 22 patients monitored other molecular markers regularly before and after allo-HSCT (*TCR*: 7, *EVI1*: 2, *E2A-PBX1*: 6, *SET-NUP214*: 1; *SIL-TAL1*: 3, *MLL*: 3), and 11 of them showed *WT1* and these molecular makers positive simultaneously before IFN-α treatment (*TCR*: 5, *EVI1*: 1, *E2A-PBX1*: 2, *SET-NUP214*: 1; *SIL-TAL1*: 1, *MLL*: 1). The cycles of IFN-α treatment was 2 (range 0.5–14) cycles, and 3 patients received IFN-α treatment for more than 6 cycles. The reasons for discontinuing IFN-α treatment included MRD turned negative (n = 21), grade ≥ 3 toxicities (infectious: n = 3; hematologic: n = 3; pulmonary: n = 1), GVHD (n = 28), and relapse (n = 12). The duration of follow-up after IFN-α treatment was 953 (range 63–1639) days.

**Table 1:** Patient characteristics between IFN-α group in the present study and non-IFN-α groups in the historical cohort.

Characteristics	IFN-α group (*n* = 68)	Non-IFN-α group (n = 18)	*P* value
Sex, male/female, *n*	44/24	11/7	0.778
Median age at allo-HSCT, years (range)	23 (9–54)	25 (7–45)	0.975
Median WBC at diagnosis, × 10^9^/L (range)	13 (1–647)	13 (1–256)	0.766
Median time from diagnosis to allo-HSCT, months (range)	6 (3–48)	6 (4–36)	0.271
First CR induction courses, *n* (%)			0.118
1	54 (79.4)	18 (100.0)	
> 1	14 (20.6)	0 (0.0)	
Pre-HSCT cycles of chemotherapy, courses (range)	4 (2–23)	7 (3–17)	< 0.001
Median time from allo-HSCT to MRD positivity, days (range)	166 (26–735)	130 (38–586)	0.811
Time from allo-HSCT to MRD positivity, *n* (%)			0.602
Early-onset MRD	22 (32.4)	7 (38.9)	
Late-onset MRD	46 (67.6)	11 (61.1)	
Median time from allo-HSCT to IFN-α treatment, days (range)	193 (36–748)	–	
Median time from MRD to IFN-α treatment, days (range)	13 (0–147)	–	
Lineage, *n* (%)			0.001
B	47 (69.1)	5 (27.8)	
T	21 (30.9)	13 (72.2)	
Cytogenetics, *n* (%)			0.651
11q23	4 (5.9)	2 (11.1)	
At least five abnormalities	7 (10.3)	1 (5.6)	
Low hypodiploidy-near triploidy	3 (4.4)	1 (5.6)	
High hyperdiploidy	2 (2.9)	1 (5.6)	
t(1;19)	1 (1.5)	0 (0.0)	
Other abnormalities	5 (7.4)	3 (16.6)	
Normal	46 (67.6)	10 (55.5)	
Disease status at allo-HSCT, *n* (%)			0.709
CR1	59 (86.8)	15 (83.3)	
CR2	9 (13.2)	3 (16.7)	
Disease risk index before allo-HSCT, *n* (%)			0.709
Intermediate risk	59 (86.8)	15 (83.3)	
High risk	9 (13.2)	3 (16.7)	
Donor–recipient relationship, *n* (%)			0.087
Mother–child	5 (7.4)	4 (22.2)	
Others	63 (92.6)	14 (77.8)	
Donor-recipient sex matched, *n* (%)			0.747
Female to male	13 (19.1)	4 (22.2)	
Others	55 (80.9)	14 (77.8)	
Donor type			0.735
HLA-identical sibling donor	12 (17.6)	4 (22.2)	
HLA-haploidentical related donor	56 (82.4)	14 (77.8)	
Number of HLA-A, -B, -DR mismatches, *n* (%)			0.222
0–1	15 (22.1)	7 (38.9)	
2–3	53 (77.9)	11 (61.1)	
Graft type, *n* (%)			–
Bone marrow and peripheral blood	68 (100.0)	18 (100.0)	
MRD status after allo-HSCT, *n* (%)			0.057
PCR positive once	29 (42.6)	3 (16.7)	
PCR positive twice	18 (26.5)	6 (33.3)	
MFC positive once	5 (7.4)	0 (0.0)	
MFC positive twice	5 (7.4)	1 (5.6)	
PCR positive and MFC positive simultaneously	11 (16.1)	8 (44.4)	
MRD level, *n* (%)			0.778
Low level	24 (35.3)	7 (38.9)	
High level	44 (64.7)	11 (61.1)	
Discontinuing immunosuppressant before IFN-α treatment, *n* (%)	46 (67.6)	–	

*allo-HSCT* allogeneic hematopoietic stem cell transplantation, *HLA* human leukocyte antigen, *IFN-α* interferon-α, *MFC* multiparameter flow cytometry, *MRD* minimal residual disease, *PCR* polymerase chain reaction, *WBC* white blood cell.

Statistical significance was set at *P* < 0.05.

### GVHD

Ten patients had aGVHD after IFN-α treatment (Table 2). The cumulative incidence of total and severe aGVHD (≥ grade III) at 4 years after treatment was 14.7% (95% CI 6.2–23.2%) and 2.9% (95% CI 0.0–6.9%), respectively.

**Table 2:** Characteristics of aGVHD after preemptive IFN-α treatment.

Characteristics of aGVHD	IFN-α group (*n* = 68)
Time from aGVHD to immunotherapy, days (range)	12 (1–64)
**Severity of aGVHD,** ***n* (%)**	
None	58 (85.3)
Grade I	3 (4.4)
Grade II	5 (7.4)
Grade III	2 (2.9)
**Site of aGVHD, *n* (%)**	
Skin	9 (13.2)
Liver	0 (0.0)
Gut	4 (5.9)
**Number of sites, *n* (%)**	
0	58 (85.3)
1	7 (10.3)
2	3 (4.4)

Data was present as *n* (%) or median (range).

*aGVHD* acute graft-versus-host disease, *IFN-α* interferon-α.

Twenty-seven patients had cGVHD after IFN-α treatment (Table 3). The cumulative incidence of total and severe cGVHD at 4 years after IFN-α treatment was 40.0% (95% CI 28.2–51.8%) and 7.5% (95% CI 1.1–13.9%), respectively.

**Table 3:** Characteristics of cGVHD after preemptive IFN-α treatment.

Characteristics of cGVHD	IFN-α group (*n* = 68)
Time from cGVHD to immunotherapy, days (range)	43 (1–404)
**Severity of cGVHD,** ***n* (%)**	
None	41 (60.3)
Mild	10 (14.7)
Moderate	12 (17.6)
Severe	5 (7.4)
**Type of cGVHD,** ***n* (%)**	
None	41 (60.3)
Classical cGVHD	24 (35.3)
Overlap syndrome	3 (4.4)
**Site of cGVHD, *n* (%)**	
Skin	21 (30.9)
Mouth	8 (11.8)
Eye	4 (5.9)
Liver	7 (10.3)
Gut	5 (7.4)
Lung	5 (7.4)
**Number of sites,** ***n* (%)**	
0	41 (60.3)
1	14 (20.6)
2	7 (10.3)
≥ 3	6 (8.8)

Data was present as *n* (%) or median (range).

*cGVHD* chronic graft-versus-host disease, *IFN-α* interferon-α.

### MRD evolution and relapse

After preemptive IFN-α treatment, 24 (35.3%), 5 (7.4%), 6 (8.8%), and 13 (19.1%) patients achieved MRD negativity at 1, 2, 3, and > 3 months, respectively, and 6 of them showed relapse. More than 80% of the patients with MRD_sin+_ achieved MRD negativity after IFN-α treatment, which was higher than that of the patients with MRD_co+_ (82.4% vs. 58.8%, *P* = 0.033). Twenty (29.4%) patients did not achieve MRD negativity after IFN-α treatment, and 15 of them showed relapse. The rate of achieving MRD negativity was 65.2% and 81.8% for the patients who discontinued and did not discontinue immunosuppressive agents before IFN-α treatment (*P* = 0.160). The cumulative incidences of achieving MRD negativity at 3 months, 6 months, 12 months, and 24 months after IFN-α treatment were 48.8% (95% CI 36.7–60.9%), 59.4% (95% CI 47.5–71.3%), 65.7% (95% CI 54.0–77.4%), and 70.7% (95% CI 59.3–82.1%), respectively.

**Figure 1:**
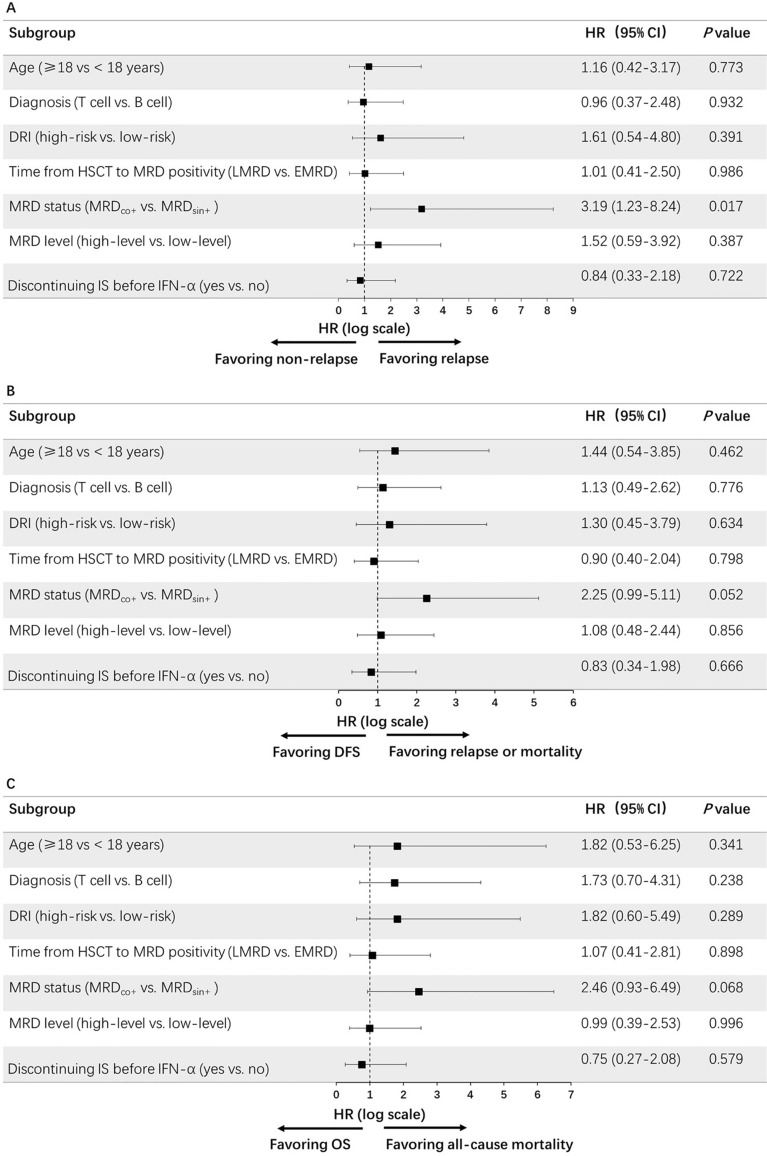
Univariate analysis for prognostic factors of preemptive IFN-α treatment: (**A**) relapse; (**B**) disease-free survival, and (**C**) overall survival.

Twenty-one patients experienced relapse after IFN-α treatment. The duration from IFN-α treatment to relapse was 110 (range 14–890) days. The cumulative incidence of relapse (CIR) at 4 years after IFN-α treatment was 31.9% (95% CI 20.5–43.3%). The 4-year CIR after IFN-α treatment was higher in the MRD_co+_ group compared to that of the MRD_sin+_ group (47.4% vs. 17.8%, *P* = 0.011). The MRD_sin+_ status before IFN-α treatment was the only factor which was associated with a low risk of relapse in univariate analysis (Fig. 1A).

### NRM

Four patients died of NRM (Supplementary Table 3). The duration from IFN-α treatment to NRM was 112 (range 77–575) days. The cumulative incidence of NRM at 4 years after IFN-α treatment was 6.0% (95% CI 3.1–8.9%).

### Survival

The probability of DFS at 4 years after IFN-α treatment was 62.1% (95% CI 50.2–74.0%). The probability of OS at 4 years after IFN-α treatment was 71.1% (95% CI 60.0–82.2%). The MRD_sin+_ status before IFN-α treatment tended to be associated with a better DFS and OS in univariate analysis (Fig. 1B, C).

### Clinical outcomes of MRD-positive patients receiving preemptive Chemo-DLI

We also analyzed the data of patients who received preemptive Chemo-DLI during the same period (Supplementary Table 1 and Supplementary Fig. 1). The median cycle of Chemo-DLI was 1 (range 1–2 cycles), and 3 patients received Chemo-DLI for more than 1 cycle. The characteristics of patients in the Chemo-DLI group were summarized in Supplementary Table 2. The characteristic of aGVHD and cGVHD after Chemo-DLI were showed in Supplementary table 4and Supplementary table 5, respectively. Twelve patients experienced relapse after Chemo-DLI and the median time from Chemo-DLI to relapse was 42 (range 9–1027) days. The 4-year CIR after Chemo-DLI was 60.1% (95% CI 48.1–72.1%). No patients died of NRM after Chemo-DLI. The probabilities of DFS and OS at 4 years after Chemo-DLI were 39.9% (95% CI 16.5–63.3%) and 67.4% (95% CI 44.7–90.1%), respectively.

### Patients receiving preemptive IFN-α treatments had better survival than those without preemptive interventions in the historical cohort

**Figure 2:**
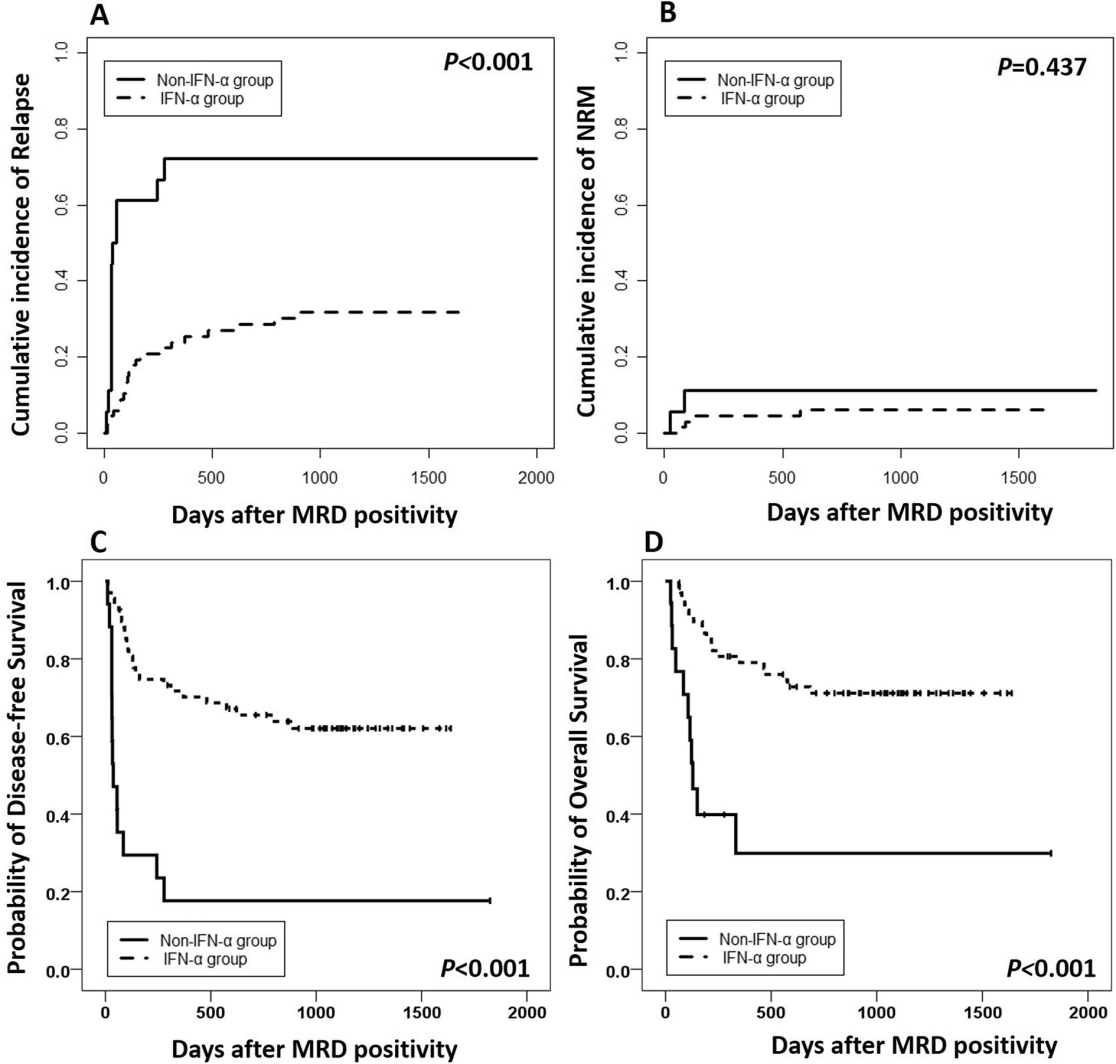
Cumulative incidence of survival after MRD positivity between the preemptive IFN-α treatment group in the present study and those who had MRD but did not receive interventions in the historical cohort: (**A**) relapse; (**B**) non-relapse mortality; (**C**) disease-free survival, and (**D**) overall survival.

To further confirm the efficacy of preemptive IFN-α treatment, a historical cohort between March 1, 2009 and May 31, 2013 including MRD-positive patients without any interventions was enrolled as controls (n = 18)^22^. T-ALL was more common in historical cohort and they had more cycles of chemotherapy before transplantation; however, the other patient characteristics were comparable between the present and the historical cohorts (Table 1). The cumulative incidences of relapse and survival were worse in the historical cohort than those receiving preemptive IFN-α treatment in the present study (Fig. 2A, C, D), and the cumulative incidence of NRM rates were comparable between the present and the historical cohorts (Fig. 2B). After adjusted by the MRD status, preemptive IFN-α treatment could also decrease the risk of relapse and improve survival (Supplementary table 6).

**Figure 3:**
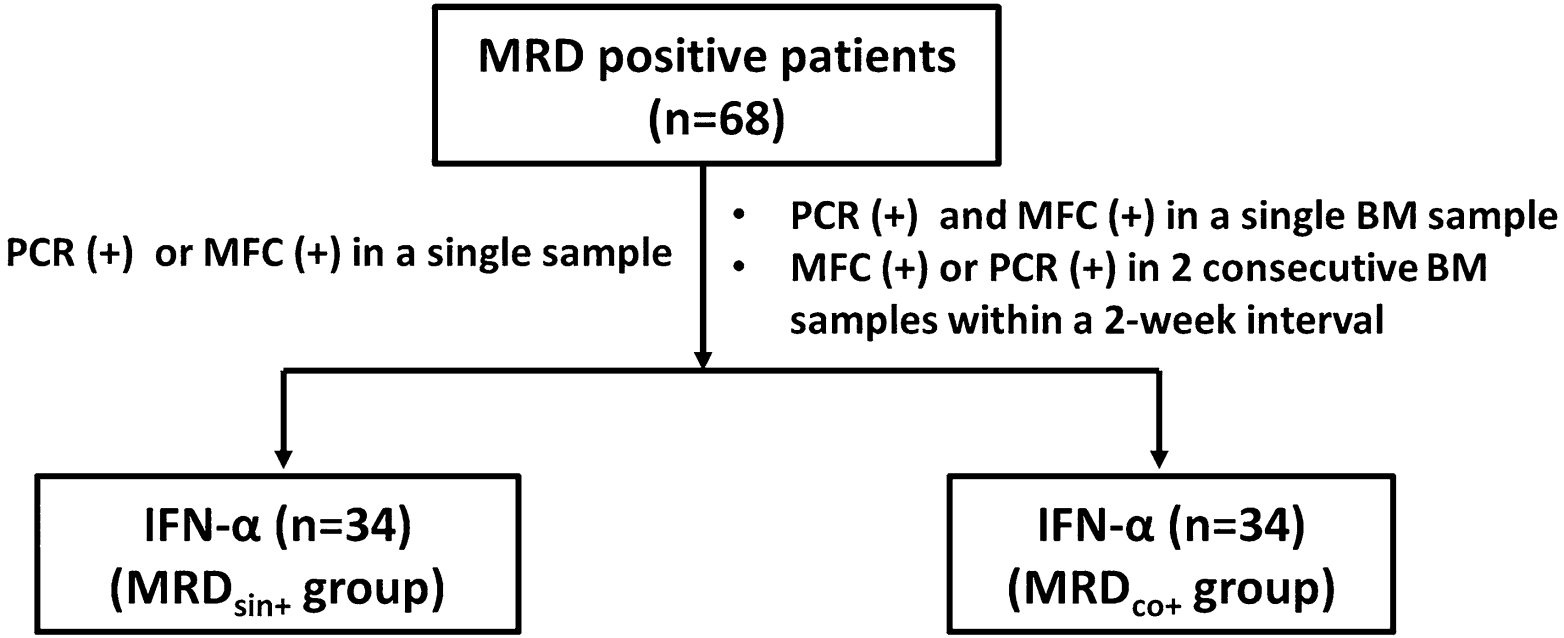
Diagram of patients enrolled.

### Clinical outcomes of patients receiving prolonged IFN-α treatment

Three patients received IFN-α treatment for more than 6 cycles at the request of themselves, 2 received 7 cycles and 1 received 14 cycles IFN-α treatment. They achieved MRD negative at 84 days, 148 days, and 396 days after IFN-α treatment, respectively. The relapse, NRM, DFS, and OS rates were 0.0% versus 31.9% (*P* = 0.272), 0.0% versus 6.0% (*P* = 0.642), 100.0% versus 62.1% (*P* = 0.230), and 100% versus 71.1% (*P* = 0.301), respectively, for those with and without prolonged IFN-α treatment.

## Discussion

In our study, the cumulative incidence of relapse and NRM at 4 years after preemptive IFN-α treatment were 31.9% and 6.0%, respectively; and the probability of DFS and OS at 4 years after preemptive IFN-α treatment were 62.1% and 71.1%, respectively. Our study is the first to study the efficacy of preemptive IFN-α treatment in a disease-specific population of patients with ALL. These results identify the undefined role of this intervention strategy in ALL patients following allo-HSCT.

The graft-versus-leukemia (GVL) effect had been described in ALL since 1970s^24^, which was further supported by a large scale study recently^25^. IFN-α can exert an immunomodulatory effect, promote the GVL effect, and clear MRD after allo-HSCT^4,18,21^. Moreover, it also showed the growth-inhibitory or cytotoxic effects on human ALL cell in vitro^26–28^. Based on these results, IFN-α emerges as a useful agent which can clear MRD through different mechanisms. In fact, IFN-α had been used as adjuvant^29–31^ or maintenance treatments in ALL patients^32,33^, which was reported to help to achieve CR again in ALL patients who experienced relapse after allo-HSCT^34,35^. Sumi et al.^33^ also reported that IFN-α helped to achieve sustained molecular CR in an ALL patient with continuing detection of MRD following allo-HSCT. However, the evidences of IFN-α as a treatment option for ALL was generally derived from single case report or small sample studies, and its clinical utility in ALL has not been consistently established. In the present study, we observed that more than 70% of the patients achieved MRD negativity after preemptive IFN-α treatment. Up to now, this is the largest study confirming that IFN-α can indeed induce clinically relevant anti-leukemic responses in ALL patients.

MFC relying on the identification of cells with LAIPs and is widely believed to be sensitive for relapse prediction in ALL patients^3,36–38^. Thus, a patient was considered as MRD_sin+_ status when a single BM sample was tested positive by MFC for LAIPs in our study. In addition, the relapse rate of ALL patients who had one positive LAIPs result was 80.0% after allo-HSCT^39^.

Approximately 60% of the patients used *WT1* as an MRD marker in the present study. *WT1* is still an important genetic marker for ALL patients^40–43^. In addition, Zhao et al.^39^ reported that the relapse rate of ALL patients who had one positive *WT1* result after allo-HSCT was 63.9%, and the sensitivity and specificity of *WT1* was 62.2% and 90.6%, respectively, for indicating ALL relapse in allo-HSCT recipients. Thus, it is reasonable to use *WT1* as the triggering marker for preemptive IFN-α treatment in ALL patients. However, some authors suggested that the sensitivity and specificity of *WT1* monitoring might be relatively low^44,45^. Thus, MFC was used in the detection of MRD simultaneously, compensating for the relatively low sensitivity of *WT1* expression. On the other hand, *WT1* was not a specific molecular marker of leukemia. It is inevitable that some patients may receive IFN-α treatment because of high *WT1* expressions which were actually not relevant to leukemia (i.e., receiving prophylactic IFN-α treatment), but Klingemann et al.^46^ demonstrated that even prophylactic IFN-α treatment could also decrease the risk of relapse after allo-HSCT. In addition, only few severe toxicities were observed during IFN-α treatment, which might minimize the impact of the relatively low specificity of *WT1* expression.

The 4-year CIR of patients who had MRD_sin+_ after preemptive IFN-α treatment was only 17.8% in the present study. Thirty-five patients with MRD_sin+_ were tested repeatedly 2 weeks after obtaining the first positive results. Among the 35 patients who showed MRD_sin+_ but did not receive IFN-α treatment, although immunosuppressions were tapered in 21 patients, only 1 patient achieved MRD negativity and the other 34 patients were tested positive for 2 consecutive BM samples (i.e., MRD_co+_). This indicated that MRD_sin+_ and MRD_co+_ might be different stages of the ALL progression. Reducing immunosuppression alone could not clear the MRD effectively and the preemptive IFN-α treatment for MRD_sin+_ patients could help to control the disease more timely. In addition, Zhao et al.^39^ reported that patients with MRD_co+_ had higher relapse rate (*WT1* + twice: 100%; MFC + twice: 87.5%; MFC + and *WT1*+: 100%) compared to that of MRD_sin+_ (*WT1* + once: 63.9%; MFC + once: 80.0%), suggesting that MRD_co+_ may represent a higher risk of relapse compared with MRD_sin+_. Our results also showed that the clinical outcomes seemed to be better in the MRD_sin+_ group than the MRD_co+_ group among patients receiving preemptive IFN-α treatment. Thus, preemptive IFN-α treatment may not completely overcome the poor prognostic significance of MRD_co+_ status of ALL, and patients with MRD_sin+_ may benefit more from preemptive IFN-α treatment after allo-HSCT.

We previously reported that preemptive Chemo-DLI could significantly decrease relapse and improve survival of patients with MRD^7^. In this study, the 4-year CIR, DFS, and OS rate of Chemo-DLI were 60.1%, 39.9%, and 67.4%, respectively. However, approximately 40% of our patients received preemptive IFN-α treatment for MRD_sin+_ and most of them could clear the MRD. These patients would not be classified as MRD_co+_ and they did not need to receive Chemo-DLI. Thus, it would be premature to derive conclusions regarding the superiority of IFN-α treatment over Chemo-DLI in patients with MRD.

A limitation to our current study was that it was not a randomized trial and the number of patients in historical control was relatively small. In addition, the ratio of PCR positive and MFC positive simultaneously seemed to be higher in non-IFN-α group although *P* value was 0.057, which meant that the risk of relapse may not be totally equivalent between IFN group and non-IFN-α group. In future, prospective, randomized trial may further confirm the efficacy of preemptive IFN-α in these patients. Secondly, the sensitivity of PCR for *WT1* transcript and MFC for LAIPs was only 10^−3^–10^−4^. With a deep detection limit and high specificity, next-generation sequencing for MRD may represent a promising tool for the ALL patients^47^, and it may further improve the efficacy of preemptive IFN-α treatment. Thirdly, IFN-α can exert anti-leukemia effect through activating NK cells; however, we did not examine the number of NK cells in the present study, and we would identify the association between the number of NK cells and MRD negativity in our future study. Lastly, besides of the CAR-T therapy, several monoclonal antibodies (MoAbs) can also target certain surface antigens on ALL cells resulting in their destruction. However, the efficacy of these MoAbs in allo-HSCT recipients with MRD was unclear. Our future prospective studies can further compare the efficacy among MRD-directed preemptive Chemo-DLI, IFN-α treatment, MoAbs, and CAR-T therapy in ALL patients following allo-HSCT^48–50^.

In conclusion, preemptive IFN-α treatment could protect against relapse and improved long-term survival of ALL patients who had MRD after allo-HSCT. Because IFN-α may tend to be started in patients with relatively low leukemia burden^17^, it could not only unlock its therapeutic potential in ALL, but also spare the patients in remission from further therapy. Moreover, IFN-α is a simple treatment with increased accessibility as it could be performed on an outpatient basis. Based on our results, future randomized clinical trials are needed to further compare the efficacy of preemptive IFN-α treatment and cytotherapies in ALL patients who had MRD after allo-HSCT.

## Patients and methods

### Patients

From June 1, 2014 to December 31, 2017, consecutive Ph-negative ALL patients receiving allo-HSCT at the Peking University Institute of Hematology (PUIH) and showed MRD positivity were enrolled if they met the following criteria: (1) ALL defined as first or second complete remission (CR) without t(9;22) mutations (Supplementary Table 1)^51^; (2) regained MRD positivity after allo-HSCT. The patients who had active GVHD, active infections, severe myelosuppression, and organ failure were excluded (Supplementary methods)^4^. Considering the probable synergistic effect between IFN-α treatment and Chemo-DLI, the patients who received both Chemo-DLI and IFN-α treatment were excluded in this study (Supplementary method; Supplementary Fig. 1). The final follow-up visits for endpoint analysis were conducted on December 31, 2019. Thirty-three patients were previously reported in 2017^4^, and all of them were enrolled and followed further in this study. The study was performed in accordance with the Declaration of Helsinki and was approved by the Ethics Committee of Peking University People’s Hospital. All patients or the patients’ guardians gave written informed consent before enrollment. The study was registered at https://clinicaltrials.gov as #NCT02185261.

### Transplant regimens

The major preconditioning regimen consisted of cytarabine (Ara-C), busulfan, cyclophosphamide, and semustine. Human leukocyte antigen (HLA)-haploidentical related donor (haplo-RD) and HLA-unrelated donor (URD) groups received rabbit anti-thymocyte globulin. All patients received granulocyte colony-stimulating factor (G-CSF)-mobilized, fresh, and unmanipulated bone marrow cells plus peripheral blood stem cells in the present study (Supplementary methods)^52–57^.

### MRD monitoring after allo-HSCT

Routine MRD monitoring was performed 1, 2, 3, 4.5, 6, 9, and 12 months post-transplantation and at 6-month intervals thereafter. MFC for LAIPs and TaqMan-based reverse transcription-real time PCR for the expressions of Wilms’ tumor gene 1 (*WT1*) were performed in all patients as a routine clinical test on bone marrow (BM) aspirate samples (Supplementary methods)^58^. When a single BM sample was tested positive by PCR or MFC, we considered this patient to have an MRD-positive status because the use of multiple methods could ensure sensitivity and specificity in the detection of the MRD^39^.

Cases in which a single BM sample was tested positive by PCR or MFC were defined as the MRD_sin+_ group. Cases in which 2 consecutive BM samples within a 2-week interval were tested positive by PCR or MFC or those in which a single BM sample was tested positive by both PCR and MFC were defined as the MRD_co+_ group (Supplementary Fig. 1).

Patients in the MRD_sin+_ group were recommended to receive preemptive IFN-α treatment. For the patients with MRD_co+_, the efficacy of Chemo-DLI had been confirmed^7^ but the role of IFN-α treatment was undefined when this study started. Thus, preemptive Chemo-DLI was the first choice for patients with MRD_co+_, and those who were unable to receive Chemo-DLI (e.g., patient or provider refusal) could receive IFN-α treatment (Fig. 3; Supplementary methods; Supplementary Table 2; Supplementary Fig. 1).

### Preemptive IFN-α treatment and Chemo-DLI protocol

The detailed protocols for preemptive IFN-α treatment and Chemo-DLI was according to the routine protocols of PUIH which had been described in detailed (Supplementary methods)^4,7,21,22^. In brief, recombinant human IFN-α-2b injections (Anferon; Tianjin Hualida Biotechnology Co., Ltd., Tianjin, China) were administered subcutaneously for 6 cycles (twice weekly in every 4 weeks cycle), at dosages of 3 million units for patients older than 16 years and at 3 million units per square meter for those younger than 16 years (capped by 3 million units). Prolonged treatment with IFN-α was permitted at the request of patients. MRD status was monitored 1, 2, 3, 4.5, 6, 9, and 12 months after preemptive IFN-α treatment and at 6-month intervals thereafter. The patients who had persistent and increasing levels of MRD or those regained MRD positivity after achieving MRD negativity after IFN-α treatment could receive salvage Chemo-DLI (Supplementary methods)^7^.

### Treatment of GVHD after preemptive immunotherapy

The treatments of acute GVHD (aGVHD) and chronic GVHD (cGVHD) were according to accepted international criteria (Supplementary methods)^59–61^.

### Definitions and assessments

Disease risk index (DRI) before allo-HSCT was described according to the criteria of Armand et al. (i.e., ALL patients in CR1 were categorized into intermediate risk, and ALL patients in CR2 were categorized into high risk group)^62^. GVHD was diagnosed according to accepted international criteria^63,64^. Definition of relapse, non-relapse mortality (NRM), early-onset MRD (EMRD), late-onset MRD (LMRD), high-level MRD, and low-level MRD were described in supplementary method^4^.

### Statistical analysis

The primary endpoint was relapse, and secondary endpoints were NRM, disease-free survival (DFS), and overall survival (OS). This study was planned to detect a relapse rate of 55% in patients with MRD receiving preemptive IFN-α treatment, from the reference rate of 75% in patients with PCR or MFC positivity but did not receive interventions in our previous study, controlling for type I and II error rates at 5% and 10%, respectively. Considering an expulsion rate of 15%, a total of 68 patients was planned to be enrolled.

Comparisons of patient characteristics between the groups were performed using the Mann–Whitney *U*-test for continuous variable and *χ*^2^ and Fisher’s exact tests for categorical data. The probability of survival was calculated using the Kaplan–Meier estimator. The incidences of GVHD were calculated using the cumulative incidence function, with death and relapse as competing risks^65^. Cumulative incidences were estimated for NRM and relapse, to account for competing risks. Relapse was the competing event for NRM and vice versa. Hazard ratios (HRs) for clinical outcomes were estimated from Cox regression analyses. *P* values were 2-sided. The SPSS 24 (SPSS Inc./IBM, Armonk, NY, USA) and the R software package (version 2.6.1; https://www.r-project.org) were used for data analyses.

### Compliance with ethics guidelines

Sining Liu, Xueyi Luo, Xiaohui Zhang, Lanping Xu, Yu Wang, Chenhua Yan, Huan Chen, Yuhong Chen, Wei Han, Fengrong Wang, Jingzhi Wang, Kaiyan Liu, Xiaojun Huang, and Xiaodong Mo declared no potential financial conflict of interest related to this manuscript. Informed consent was obtained from all patients or their guardians. The study was conducted in accordance with the Declaration of Helsinki. The study protocol was approved by the Ethics Committee of Peking University People’s Hospital.

## Supplementary information

Supplementary information 1.

Supplementary information 2.

Supplementary information 3.

Supplementary Figure 1 Legend.

Supplementary Figure 1.
