# Supplementary material for: Preemptive interferon-α treatment could protect against relapse and improve long-term survival of ALL patients after allo-HSCT
Source: Sci Rep. 2020 Nov 19;10:20148. doi: 10.1038/s41598-020-77186-9 (PMC7677364; doi:10.1038/s41598-020-77186-9)
Supplement: Supplementary file 1 — Supplementary information 1. [file 41598_2020_77186_MOESM1_ESM.docx]

**Title:** **Preemptive interferon-α treatment could protect against relapse and improve long-term survival of ALL patients after allo-HSCT.**

**Authors:** Sining Liu^1^, Xueyi Luo^1^, Xiaohui Zhang^1^, Lanping Xu^1^, Yu Wang^1^, Chenhua Yan^1^, Huan Chen^1^, Yuhong Chen^1^, Wei Han^1^, Fengrong Wang^1^, Jingzhi Wang^1^, Kaiyan Liu^1^, Xiaojun Huang^1,2^, and Xiaodong Mo (🖂)^1^

**Supplementary methods**

**Inclusion criteria and exclusion criteria**

***Inclusion criteria*:**

Consecutive patients subjects receiving non-T-cell–depleted allo-HSCT at the Peking University Institute of Hematology were enrolled if they met the following criteria: (1) acute lymphoblastic leukemia (ALL) defined as first or second complete remission (CR) without t(9;22) mutations; (2) regained minimal residual disease (MRD) positivity after allo-HSCT.

***Exclusion criteria*:**

Active acute GVHD (aGVHD), active chronic GVHD (cGVHD), active infections, severe myelosuppression (white blood cell count <1.0×10^9^ cells/L, absolute neutrophil count <0.5×10^9^ cells/L, hemoglobin count <65 g/L, or platelet count <25×10^9^ cells/L), and organ failure.

**Transplant regimens**

The major preconditioning consisted of cytarabine (Ara-C), busulfan (3.2 mg·kg^−1^·day^−1^ administered intravenously on days −8 to −6) (day 0 being the first day of donor cell infusion), cyclophosphamide (CY, 1.8 g·m^−2^·day^−1^, days −5 to −4), and semustine (250 mg·m^−2^, day −3). Ara-C was administered at 4 g·m^−2^·day^−1^ (days −10 to −9) to the human leukocyte antigen (HLA)-haploidentical related donor (haplo-RD) group, at 2 g·m^−2^·day^−1^ (days −10 to −9) to the HLA-unrelated donor (URD) group, and at 2 g·m^−2^·day^−1^ (day −9) to the HLA-identical sibling donor (ISD) group. Rabbit antithymocyte globulin (thymoglobulin, 2.5 mg·kg^−1^·day^−1^, days −5 to −2; Sanofi, France) was administered to the haplo-RD and URD groups. One patient received a conditioning regimen including total body irradiation (TBI). Granulocyte colony-stimulating factor (G-CSF)-mobilized, fresh, and unmanipulated bone marrow (BM) and peripheral blood harvests were infused into the recipients on the day of collection. In addition, patients received cyclosporine A (CSA), mycophenolate mofetil (MMF), and short-term methotrexate (MTX) as GVHD prophylaxis.

**MRD monitoring and definition**

MRD was monitored according to leukemia-associated aberrant immune phenotypes (LAIPs) and Wilms’ tumor gene 1 (*WT1*) in patients with acute leukemia. In brief, multiparameter flow cytometry (MFC) was performed in all patients as a routine clinical test on bone marrow aspirate samples. A panel of eight antibody combinations that recognize cCD3, mCD3, CD2, CD5, CD7, CD10, CD19, CD20, CD34, CD38, CD45, CD58, CD99, CD123, and cTDT was used for ALL-LAIP detection. The isotype control monoclonal antibodies were used, and 0.2-1 million events per tube were acquired on a FACS Cant II. Positive MRD was considered when a cluster of more than 20 cells with LAIP and SSC characteristics, identified in all plots of interest and carrying at least two LAIP markers identified at diagnosis, was observed. For those without LAIP markers at diagnosis, MRD was identified as a cell population showing deviation from the normal patterns of antigen expression seen on specific cell lineages at specific stages of maturation compared with either normal or regenerating marrow. The expressions of *WT1* were evaluated by TaqMan-based real time quantitative reverse transcription polymerase chain reaction (RQ-PCR). We selected ABL as a control gene. The experiments were performed in duplicate. The transcript level was calculated as *WT1* transcript copies/ABL copies in percentage. *WT1* transcript level >0.60% was defined as positive. Routine MRD monitoring was performed 1, 2, 3, 4.5, 6, 9, and 12 months post-transplantation and at 6-month intervals thereafter. Because we demonstrated that the combined use of PCR and MFC might achieve higher sensitivity without sacrificing specificity, a patient was considered to have an MRD-positive status when a single BM sample tested positive for MFC or PCR.

**Preemptive IFN-α treatment protocol**

Cases in which a single BM sample tested positive for PCR or MFC were defined as MRD_sin+_ group. Cases in which 2 consecutive BM samples within a 2-week interval tested positive for PCR or MFC or those in which a single BM sample tested positive for both PCR and MFC were defined as MRD_co+_ group.

Patients with MRD_sin+_ were recommended to receive IFN-α treatment. For the patients who did not agree to receive IFN-α treatment, the tests were repeated 2 weeks after positive results for PCR or MFC results were obtained (reducing immunosuppression alone was not considered as preemptive intervention in the present studies; n=21). If 2 consecutive BM samples tested positive for PCR or MFC (MRD_co+_) within a 2-week interval, patients should receive preemptive intervention. In addition, cases in which a single BM sample tests positive for both PCR and MFC (MRD_co+_) should also receive preemptive intervention. Because the efficacy of Chemo-DLI had been confirmed but the role of IFN-α treatment was still undefined in MRD_co+_ patients when these two studies started, preemptive Chemo-DLI was the first choice for patients in MRD_co+_ group, and those who could not receive Chemo-DLI because of patient or provider refusal received IFN-α treatment and enrolled in these two studies.

Recombinant human IFN-α-2b injections (Anferon; Tianjin Hualida Biotechnology Co., Ltd., Tianjin, China) were administered subcutaneously for 6 cycles (twice weekly in every 4 weeks cycle) at dosages of 3 million units for patients older than 16 years, and at 3 million units per square meter for those younger than 16 years (capped by 3 million units). Prolonged treatment with IFN-α was permitted at the request of patients. MRD status was monitored 1, 2, 3, 4.5, 6, 9, and 12 months after preemptive IFN-α treatment and at 6-month intervals thereafter. Adverse events were scored using the National Cancer Institute Common Toxicity Criteria version 4.0, and they were monitored every 1-2 weeks after IFN-α treatment. GVHD was excluded as an adverse event. Study medication with IFN-α was discontinued in any patient with active GVHD (grade II or higher aGVHD or cGVHD with moderate or higher severity), severe infection, grade ≥ 3 toxicity, salvage Chemo-DLI use, relapse, or non-relapse mortality (NRM).

The patients who showed unsatisfactory response to IFN-α treatment can receive salvage Chemo-DLI, if they agreed to receive Chemo-DLI and did not have active GVHD, active infection, and organ failure were eligible for salvage Chemo-DLI: patients had positive MRD again after achieving MRD-negative status (n=3) or those with persistent and increasing level of MRD (e.g., rising from low-level MRD to high-level MRD; n=12) after preemptive IFN-α treatment. All the 15 patients received salvage Chemo-DLI before impending relapse, and they were not enrolled in the analysis of the present study.

**Preemptive Chemo-DLI protocol**

G-CSF–mobilized peripheral blood stem cells were administered instead of the more common unstimulated donor blood lymphocytes. Patients also received anti-leukemic chemotherapy 48–72 hours before DLI. Chemotherapy regimens included MTX (1.0 g∙m^−2^∙day^−1^ for 1 day, n=14) or CODP (CY 800 mg∙m^−2^∙day^−1^ for 2 days, vincristine 1 mg∙m^−2^∙day^−1^ for 1 day, daunorubicin 40 mg∙m^−2^∙day^−1^ for 3 days, and prednisone 60 mg^.^day^-1^ for 7 days, n=7).

G-CSF–mobilized peripheral leukocytes were administered instead of the unstimulated donor blood lymphocytes. The dose of mononuclear cells was 1.0 × 10^8^/kg, and the median doses of CD3^+^ cells and CD34^+^ cells were 3.7 (1.5–5.7) × 10^7^/kg, and 0.4 (0.1–0.9) × 10^6^/kg, respectively.

Patients received immunosuppressive drugs such as CSA (n=20) or MTX (n=1) to prevent GVHD after DLI. Patients receiving DLI from an ISD received GVHD prophylaxis for 4–6 weeks, while those receiving DLI from a haplo-RD or URD received GVHD prophylaxis for 6–8 weeks at the discretion of the attending physicians (and usually depending on the patient’s GVHD status after Chemo-DLI). The starting dosage of CSA was 2.5 mg·kg^−1^·day^−1^, which was adjusted to maintain a plasma concentration >100 ng/mL. MTX was administered at 10 mg intravenously on days 1, 4, 8, and weekly thereafter for 2–6 weeks. MRD status was monitored 1, 2, 3, 4.5, 6, 9, and 12 months after preemptive Chemo-DLI and at 6-month intervals thereafter.

**Treatment of GVHD after preemptive intervention**

aGVHD was treated with methylprednisolone (1–2 mg∙kg^-1^ per day) and by resumption of full-dose CSA administration. Second- or third-line immunosuppressive therapies such as CD25 monoclonal antibody (Basiliximab; Novartis Pharma Stein AG, Basel, Switzerland), MMF, tacrolimus, or MTX were administered in cases of steroid-refractory aGVHD. Moderate to severe cGVHD was treated with prednisone (1 mg∙kg^-1^ per day), and CSA was adjusted to maintain a trough blood concentration >150 ng/mL. Second- or third-line immunosuppressive therapies such as MMF, MTX, penicillamine, azathioprine, rituximab, or tacrolimus were administered in cases of steroid-refractory cGVHD.

**Definition**

Relapse was defined as morphologic evidence of disease in samples from the peripheral blood, bone marrow, or extramedullary sites or by the recurrence and sustained presence of pre-transplantation chromosomal abnormalities. Patients who exhibited MRD were not classified as showing relapse. NRM was defined as death without disease progression or relapse. Overall survival (OS) was defined as death from any cause. Disease-free survival (DFS) was defined as the survival period with continuous CR. Early-onset MRD (EMRD) was defined as testing positive for MRD ≤100 days after allo-HSCT, and late-onset MRD (LMRD) was defined as testing positive for MRD >100 days after allo-HSCT. High-level MRD were defined as *WT1* transcript levels ≥1.0% and/or LAIP positivity in ≥0.1% of cells with LAIPs in post-transplantation BM samples; the other cases were defined as cases with low MRD levels.
