# Supplementary material for: Preemptive interferon-α treatment could protect against relapse and improve long-term survival of ALL patients after allo-HSCT
Source: Sci Rep. 2020 Nov 19;10:20148. doi: 10.1038/s41598-020-77186-9 (PMC7677364; doi:10.1038/s41598-020-77186-9)
Supplement: Supplementary file 2 — Supplementary information 2. [file 41598_2020_77186_MOESM2_ESM.docx]

**Title:** **Preemptive interferon-α treatment could protect against relapse and improve long-term survival of ALL patients after allo-HSCT.**

**Authors:** Sining Liu^1^, Xueyi Luo^1^, Xiaohui Zhang^1^, Lanping Xu^1^, Yu Wang^1^, Chenhua Yan^1^, Huan Chen^1^, Yuhong Chen^1^, Wei Han^1^, Fengrong Wang^1^, Jingzhi Wang^1^, Kaiyan Liu^1^, Xiaojun Huang^1,2^, and Xiaodong Mo (🖂)^1^

**Supplementary table 1. Indications for allo-HSCT in CR1 patients**

| **High-risk factors** | **IFN-α group**  **(*n* =59)** | **Chemo-DLI group**  **(n=14)** | **Non-IFN-α group**  **(n=15)** |
| --- | --- | --- | --- |
| **Adults** | **52** | **10** | **12** |
| Fail to achieve CR within 28–30 days, *n* | 12 | 2 | 0 |
| MRD(+) 8 weeks after induction therapy, *n* | 22 | 8 | 2 |
| Age > 35, *n* | 8 | 3 | 3 |
| High WBC count at presentation, *n* | 9 | 3 | 0 |
| Adverse cytogenetics, *n* | 12 | 2 | 2 |
| Early/mature T-ALL, *n* | 3 | 2 | 5 |
| **Showed at least 1 high-risk factor, *n*** | **49** | **10** | **11** |
| Showed≥2 high-risk factors, *n* | 13 | 6 | 1 |
| Patients strongly demanded allo-HSCT, *n* | 3 | 0 | 1 |
| **Children** | **7** | **4** | **3** |
| Fail to achieve CR within 28–30 days, *n* | 0 | 2 | 0 |
| MRD > 0.1% within 12 weeks after therapy, *n* | 2 | 2 | 1 |
| High WBC count at presentation, *n* | 4 | 0 | 1 |
| Adverse cytogenetics, *n* | 0 | 1 | 1 |
| Early/mature T-ALL, *n* | 1 | 1 | 0 |
| **Showed at least 1 high-risk factor, *n*** | **7** | **3** | **3** |
| Showed≥2 high-risk factors, *n* | 0 | 2 | 0 |
| Parents strongly demanded allo-HSCT, *n* | 0 | 1 | 0 |

**Supplementary table 2. Patient characteristics between IFN-α and Chemo-DLI group**

| **Characteristics** | **IFN-α**  **group**  **(*n* =68)** | **Chemo-DLI group**  **(n=21)** |
| --- | --- | --- |
| Sex, male/female, *n* | 44/24 | 10/11 |
| Median age at allo-HSCT, years (range) | 23 (9–54) | 15 (7–44) |
| Median time from diagnosis to allo-HSCT, months (range) | 6 (3–48) | 8 (3–53) |
| First CR induction courses, *n* (%) |  |  |
| 1 | 54 (79.4) | 16 (76.2) |
| > 1 | 14 (20.6) | 5 (23.8) |
| Median time from allo-HSCT to MRD positivity, days (range) | 166 (26–735) | 185 (50–1112) |
| Time from allo-HSCT to MRD positivity, *n* (%) |  |  |
| Early-onset MRD | 22 (32.4) | 5 (23.8) |
| Late-onset MRD | 46 (67.6) | 16 (76.2) |
| Median time from allo-HSCT to immunotherapy, days (range) | 193 (36–748) | 196 (62–1128) |
| Median time from MRD to immunotherapy, days (range) | 13 (0–147) | 15 (5–41) |
| Lineage, *n* (%) |  |  |
| B | 47 (69.1) | 16 (76.2) |
| T | 21 (30.9) | 5 (23.8) |
| Disease status at allo-HSCT, *n* (%) |  |  |
| CR1 | 59 (86.8) | 14 (66.7) |
| CR2 | 9 (13.2) | 7 (33.3) |
| Disease risk index before allo-HSCT, *n* (%) |  |  |
| Intermediate risk | 59 (86.8) | 15 (71.4) |
| High risk | 9 (13.2) | 6 (28.6) |
| Donor–recipient relationship, *n* (%) |  |  |
| Mother–child | 5 (7.4) | 0 (0.0) |
| Others | 63 (92.6) | 21 (100.0) |
| Donor-recipient sex matched, *n* (%) |  |  |
| Female to male | 13 (19.1) | 4 (19.0) |
| Others | 55 (80.9) | 17 (81.0) |
| Donor type |  |  |
| HLA-identical sibling donor | 12 (17.6) | 6 (28.6) |
| HLA-haploidentical related donor | 56 (82.4) | 14 (66.7) |
| HLA-unrelated donor | 0 (0.0) | 1 (4.7) |
| Number of HLA-A, HLA-B, HLA-DR mismatches, *n* (%) |  |  |
| 0-1 | 15 (22.1) | 9 (42.9) |
| 2-3 | 53 (77.9) | 12 (57.1) |
| MRD status before immunotherapy, *n* (%) |  |  |
| PCR positive once | 29 (42.6) | 0 (0.0) |
| PCR positive twice | 18 (26.5) | 8 (38.1) |
| MFC positive once | 5 (7.4) | 0 (0.0) |
| MFC positive twice | 5 (7.4) | 3 (14.3) |
| PCR positive and MFC positive simultaneously | 11 (16.1) | 10 (47.6) |
| MRD level before immunotherapy, *n* (%) |  |  |
| Low level | 24 (35.3) | 4 (19.0) |
| High level | 44 (64.7) | 17 (81.0) |
| Discontinuing immunosuppressant before immunotherapy, *n* (%) | 46 (67.6) | 13 (61.9) |
| Median duration of follow-up after immunotherapy, days (range) | 953  (63–1639) | 645  (40–2038) |

Data was present as *n* (%) or median (range).

allo-HSCT, allogeneic hematopoietic stem cell transplantation; Chemo-DLI, chemotherapy plus donor lymphocyte infusion; CR, complete remission; HLA, human leukocyte antigen; IFN-α, interferon-α; MFC, multiparameter flow cytometry; MRD, minimal residual disease; PCR, polymerase chain reaction.

Statistical significance was set at *P* < 0.05.

**Supplementary table 3 Causes of death**

| **Cause** | **IFN-α group**  **(*n* =68)** | **Chemo-DLI group**  **(n=21)** |
| --- | --- | --- |
| Relapse mortality | 15 | 6 |
| Non-relapse mortality |  |  |
| Infection | 2 | 0 |
| Graft-versus-host disease | 1 | 0 |
| Diffuse alveolar hemorrhage | 1 | 0 |
| Total | 19 | 6 |

Chemo-DLI, chemotherapy plus donor lymphocyte infusion; IFN-α, interferon-α

**Supplementary table 4 Characteristics of aGVHD after preemptive immunotherapy**

| **Characteristics of aGVHD** | **IFN-α group (*n* =68)** | **Chemo-DLI group (n=21)** |
| --- | --- | --- |
| Time from aGVHD to immunotherapy, days (range) | 12 (1–64) | 40 (15–58) |
| Severity of aGVHD, *n* (%) |  |  |
| None | 58 (85.3) | 17 (81.0) |
| Grade I | 3 (4.4) | 1 (4.8) |
| Grade II | 5 (7.4) | 2 (9.4) |
| Grade III | 2 (2.9) | 1 (4.8) |
| Site of aGVHD, *n* (%) |  |  |
| Skin | 9 (13.2) | 3 (14.3) |
| Liver | 0 (0.0) | 1 (4.8) |
| Gut | 4 (5.9) | 2 (9.5) |
| Number of sites, *n* (%) |  |  |
| 0 | 58 (85.3) | 17 (81.0) |
| 1 | 7 (10.3) | 2 (9.5) |
| 2 | 3 (4.4) | 2 (9.5) |

Data was present as *n* (%) or median (range).

aGVHD, acute graft-versus-host disease; Chemo-DLI, chemotherapy plus donor lymphocyte infusion; IFN-α, interferon-α.

**Supplementary table 5 Characteristics of cGVHD after preemptive immunotherapy**

| **Characteristics of cGVHD** | **IFN-α group (*n* =68)** | **Chemo-DLI group (n=21)** |
| --- | --- | --- |
| Time from cGVHD to immunotherapy, days (range) | 43 (1–404) | 80 (32–528) |
| Severity of cGVHD, *n* (%) |  |  |
| None | 41 (60.3) | 11 (52.4) |
| Mild | 10 (14.7) | 0 (0.0) |
| Moderate | 12 (17.6) | 3 (14.3) |
| Severe | 5 (7.4) | 7 (33.3) |
| Type of cGVHD, *n* (%) |  |  |
| None | 41 (60.3) | 11 (52.4) |
| Classical cGVHD | 24 (35.3) | 8 (38.1) |
| Overlap syndrome | 3 (4.4) | 2 (9.5) |
| Site of cGVHD, *n* (%) |  |  |
| Skin | 21 (30.9) | 7 (33.3) |
| Mouth | 8 (11.8) | 4 (19.0) |
| Eye | 4 (5.9) | 1 (4.8) |
| Liver | 7 (10.3) | 6 (28.6) |
| Gut | 5 (7.4) | 2 (9.5) |
| Lung | 5 (7.4) | 2 (9.5) |
| Number of sites, *n* (%) |  |  |
| 0 | 41 (60.3) | 11 (52.4) |
| 1 | 14 (20.6) | 4 (19.0) |
| 2 | 7 (10.3) | 2 (9.6) |
| ≥3 | 6 (8.8) | 4 (19.0) |

Data was present as *n* (%) or median (range).

cGVHD, chronic graft-versus-host disease; Chemo-DLI, chemotherapy plus donor lymphocyte infusion; IFN-α, interferon-α.

**Supplementary table 6 Unadjusted and adjusted impact of preemptive IFN-α treatment for ALL patients after allo-HSCT**

| Variable | Relapse | | Treatment failure as defined by DFS | | Treatment failure as defined by OS | |
| --- | --- | --- | --- | --- | --- | --- |
|  | HR (95% CI) | *P* value | HR (95% CI) | *P* value | HR (95% CI) | *P* value |
| Unadjusted | 0.43 (0.31–0.63) | <0.001 | 0.45 (0.32–0.62) | <0.001 | 0.47 (0.32–0.69) | <0.001 |
| Adjusted by MRD status | 0.45 (0.31–0.65) | <0.001 | 0.45 (0.32–0.64) | <0.001 | 0.50 (0.33–0.73) | <0.001 |

ALL, acute lymphoblastic leukemia; allo-HSCT, allogeneic hematopoietic stem cell transplantation; CI, confidence interval; DFS, disease-free survival; HR, hazard ratio; IFN-α, interferon-α; MRD, minimal residual disease; OS, overall survival.

Statistical significance was set at *P* < 0.05.

The analysis were adjusted by MRD status (PCR positive and MFC positive simultaneously vs. others).

None of variables was significantly associated with increased NRM in multivariate analysis.
