# Supplementary material for: Preemptive interferon-α treatment could protect against relapse and improve long-term survival of ALL patients after allo-HSCT
Source: Sci Rep. 2020 Nov 19;10:20148. doi: 10.1038/s41598-020-77186-9 (PMC7677364; doi:10.1038/s41598-020-77186-9)
Supplement: Supplementary file 3 — Supplementary information 3. [file 41598_2020_77186_MOESM3_ESM.docx]

**Supplementary methods - Trial Protocol**

**Project summary**

This study aimed to evaluate the efficacy of interferon-α (IFN-α) among acute leukemia patients who had minimal residual disease (MRD) after allogeneic hematopoietic stem cell transplantation (allo-HSCT).

Allo-HSCT is an effective treatment option for acute leukemia and many other hematological malignancies. However, post-transplant relapse is still inevitable, and the clinical outcomes of these patients are very poor. The persistence or recurrence of MRD in the post-transplant period is an independent risk factor for relapse. Therefore, MRD monitoring can be used to screen patients with a higher risk of relapse, direct timely interventions, and prevent post-transplant relapse. IFN-α-2b exerts a relatively strong immunomodulatory effect. It can kill acute leukemia (AL) cells by regulating T-cell and/or natural killer cell functions. Consequently, IFN-α-2b may have potential therapeutic value for AL patients with MRD after allo-HSCT.

The study hypothesis:

MRD directed IFN-α-2b treatment can decrease post-HSCT relapse rate of patients with standard risk AL following allo-HSCT.

**General information**

**Protocol title:** Interferon-α for the Therapy of Minimal Residual Disease Following Hematopoietic Stem Cell Transplantation

**Protocol number and date:** #NCT02185261 (01/06/2014)

**Sponsor:** Peking University People's Hospital

**Investigator: Prof** Xiaojun Huang, Peking University People’s Hospital, Peking University Institute of Hematology, No. 11 Xizhimen South Street, Xicheng District, Beijing 100044, China, Tel: 8610-8832-6006, Fax: 8610-8832-4577, e-mail: huangxiaojun@bjmu.edu.cn

**Rationale & background information**

Despite considerable advances in the allogeneic hematopoietic stem cell transplantation (allo-HSCT) ^1^, relapse remains the major cause of transplant failure in patients with acute lymphoblastic leukemia (ALL) ^2^. Thus, identifying patients who were at higher risks for relapse after allo-HSCT is of great importance. Minimal residual disease (MRD) helped to identify patients who still harbored higher levels of disease but were below the detection capabilities of morphological analysis. Multiparameter flow cytometry (MFC) identified cells with leukemia-associated immunophenotypes (LAIPs) and polymerase chain reaction (PCR) assays detected leukemia-associated genetic abnormalities, both could be applied for monitoring MRD in leukemia patients. MRD monitoring was proved to predict impending relapse after allo-HSCT by numerous studies ^3-5^.

Impending relapse could be prevented by the early detection of MRD and timely treatments. Thus, preemptive intervention, which could spare patients in remission from further therapies, was reasonable for patients with MRD. Chemotherapy plus donor leukocyte infusion (Chemo-DLI) was the most effective preemptive intervention for MRD ^6,7^, however, it may lead to some severe complications (e.g., graft-versus-host disease [GVHD] and pancytopenia) ^8^. In addition, it was out of choices for some patients because of related donor refusal or unavailability of the second donation from an unrelated donor. Preemptive tyrosine kinase inhibitor (TKIs) treatment was proved to be a useful intervention ^9,10^, but only applied to patients with Philadelphia chromosome (Ph)-positive ALL. Chimeric antigen receptor (CAR) T-cell immunotherapy was another potential preemptive intervention ^11-13^. However, it might also lead to several complications (e.g. life-threatening neurological toxicity and cytokine release syndrome) ^14,15^, and remissions after CAR T-cell treatment was relatively brief because of poor CAR T cell persistence and/or leukemia cell resistance ^16^.

Interferon-α (IFN-α) had shown activity in acute leukemia through immune activation ^17^, which rekindled the interest of using IFN-α as an immunotherapy for patients receiving allo-HSCT ^18^. Our pilot studies showed that IFN-α was a safe agent for allo-HSCT recipients ^19^. We further confirmed that preemptive IFN-α treatment can clear the MRD effectively in patients with acute leukemia and high-risk myelodysplastic syndrome after allo-HSCT ^4,20-22^. IFN-α could also be used as a salvage treatment for patients with unsatisfactory response to preemptive Chemo-DLI ^23^. However, the sample of ALL patients enrolled in these studies was relatively small, and no study had identified the efficacy of preemptive IFN-α treatment in a disease-specific population of patients with ALL after allo-HSCT. In addition, the follow-ups of these patients were short. Thus, the long-term efficacy of preemptive IFN-α treatment remains unknown in ALL patients following allo-HSCT.

Therefore, we aimed to identify the safety and long-term efficacy of preemptive IFN-α treatment in ALL patients following allo-HSCT.

**Reference**

1 Xu, L. *et al.* The consensus on indications, conditioning regimen, and donor selection of allogeneic hematopoietic cell transplantation for hematological diseases in China-recommendations from the Chinese Society of Hematology. *J Hematol Oncol* **11**, 33-33,(2018).

2 Yan, C. H. *et al.* Causes of mortality after haploidentical hematopoietic stem cell transplantation and the comparison with HLA-identical sibling hematopoietic stem cell transplantation. *Bone Marrow Transplant* **51**, 391-397,(2016).

3 Campana, D. & Pui, C. H. Minimal residual disease-guided therapy in childhood acute lymphoblastic leukemia. *Blood* **129**, 1913-1918,(2017).

4 Mo, X. D. *et al.* IFN-alpha Is Effective for Treatment of Minimal Residual Disease in Patients with Acute Leukemia after Allogeneic Hematopoietic Stem Cell Transplantation: Results of a Registry Study. *Biol Blood Marrow Transplant* **23**, 1303-1310,(2017).

5 Wang, Y. *et al.* The consensus on the monitoring, treatment, and prevention of leukemia relapse after allogeneic hematopoietic stem cell transplantation in China. *Cancer Lett* **438**, 63-75,(2018).

6 Dominietto, A. *et al.* Donor lymphocyte infusions for the treatment of minimal residual disease in acute leukemia. *Blood* **109**, 5063-5064,(2007).

7 Yan, C. H. *et al.* Risk stratification-directed donor lymphocyte infusion could reduce relapse of standard-risk acute leukemia patients after allogeneic hematopoietic stem cell transplantation. *Blood* **119**, 3256-3262,(2012).

8 Orti, G. *et al.* Donor lymphocyte infusions in AML and MDS: Enhancing the graft-versus-leukemia effect. *Exp Hematol* **48**, 1-11,(2017).

9 Chen, H. *et al.* Administration of imatinib after allogeneic hematopoietic stem cell transplantation may improve disease-free survival for patients with Philadelphia chromosome-positive acute lymphobla stic leukemia. *J Hematol Oncol* **5**, 29,(2012).

10 Wassmann, B. *et al.* Early molecular response to posttransplantation imatinib determines outcome in MRD+ Philadelphia-positive acute lymphoblastic leukemia (Ph+ ALL). *Blood* **106**, 458-463,(2005).

11 Cheng, Y. *et al.* Donor-Derived CD19-Targeted T Cell Infusion Eliminates B Cell Acute Lymphoblastic Leukemia Minimal Residual Disease with No Response to Donor Lymphocytes after Allogeneic Hematopoietic Stem Cell Transplantation. *Engineering* **5**, 150-155,(2019).

12 Ogba, N. *et al.* Chimeric Antigen Receptor T-Cell Therapy. *J Natl Compr Canc Netw* **16**, 1092-1106,(2018).

13 Park, J. H. *et al.* Long-Term Follow-up of CD19 CAR Therapy in Acute Lymphoblastic Leukemia. *N Engl J Med* **378**, 449-459,(2018).

14 Chen, H. *et al.* Management of cytokine release syndrome related to CAR-T cell therapy. *Front Med* **13**, 610-617,(2019).

15 Maude, S. L., Teachey, D. T., Porter, D. L. & Grupp, S. A. CD19-targeted chimeric antigen receptor T-cell therapy for acute lymphoblastic leukemia. *Blood* **125**, 4017-4023,(2015).

16 Shah, N. N. & Fry, T. J. Mechanisms of resistance to CAR T cell therapy. *Nat Rev Clin Oncol* **16**, 372-385,(2019).

17 Anguille, S. *et al.* Interferon-α in acute myeloid leukemia: an old drug revisited. *Leukemia* **25**, 739-748,(2011).

18 Lin, X. J. *et al.* Effects of preemptive interferon-alpha monotherapy in acute leukemia patients with relapse tendency after allogeneic hematopoietic stem cell transplantation: a case-control study. *Ann Hematol* **97**, 2195-2204,(2018).

19 Mo, X. *et al.* Interferon alpha: the salvage therapy for patients with unsatisfactory response to minimal residual disease-directed modified donor lymphocyte infusion. *Chin Med J (Engl)* **127**, 2583-2587,(2014).

20 Mo, X. *et al.* Minimal residual disease-directed immunotherapy for high-risk myelodysplastic syndrome after allogeneic hematopoietic stem cell transplantation. *Front Med* **13**, 354-364,(2019).

21 Mo, X.-D. *et al.* Interferon-α Is Effective for Treatment of Minimal Residual Disease in Patients with t(8;21) Acute Myeloid Leukemia After Allogeneic Hematopoietic Stem Cell Transplantation: Results of a Prospective Registry Study. *Oncologist* **23**, 1349-1357,(2018).

22 Mo, X.-D. *et al.* Interferon-α: A Potentially Effective Treatment for Minimal Residual Disease in Acute Leukemia/Myelodysplastic Syndrome after Allogeneic Hematopoietic Stem Cell Transplantation. *Biol Blood Marrow Transplant* **21**, 1939-1947,(2015).

23 Mo, X. *et al.* Interferon-alpha salvage treatment is effective for patients with acute leukemia/myelodysplastic syndrome with unsatisfactory response to minimal residual disease-directed donor lymphocyte infusion after allogeneic hematopoietic stem cell transplantation. *Front Med* **13**, 238-249,(2019).

**Study goals and objectives:** we aimed to identify the safety and long-term efficacy of preemptive IFN-α treatment in ALL patients following allo-HSCT. The primary objective was relapse, the secondary objective included non-relapse mortality (NRM), overall survival (OS) and disease-free survival (DFS).

**Study design**

**Study Type:** Interventional (Clinical Trial)

**Estimated Enrollment for the ALL subgroup:** 68

This study was planned to detect a relapse rate of 55% in patients with MRD receiving preemptive IFN-α treatment, from the reference rate of 75% in patients with PCR or MFC positivity but did not receive interventions in our previous study, controlling for type I and II error rates at 5% and 10%, respectively. Considering an expulsion rate of 15%, a total of 68 patients was planned to be enrolled.

**Study Start Date for ALL subgroup:** June 2014

**Study completion date for ALL subgroup:** December 2017

**Expected duration of the study:** 2 years after the enrollment of the last patients

**Inclusion criteria and exclusion criteria**

***Inclusion criteria*:**

Consecutive patients subjects receiving non-T-cell–depleted allo-HSCT at the Peking University Institute of Hematology were enrolled if they met the following criteria: (1) acute lymphoblastic leukemia (ALL) defined as first or second complete remission (CR) without t(9;22) mutations; (2) regained minimal residual disease (MRD) positivity after allo-HSCT.

***Exclusion criteria*:**

Active GVHD (aGVHD), active chronic GVHD (cGVHD), active infections, severe myelosuppression (white blood cell count <1.0×10^9^ cells/L, absolute neutrophil count <0.5×10^9^ cells/L, hemoglobin count <65 g/L, or platelet count <25×10^9^ cells/L), and organ failure


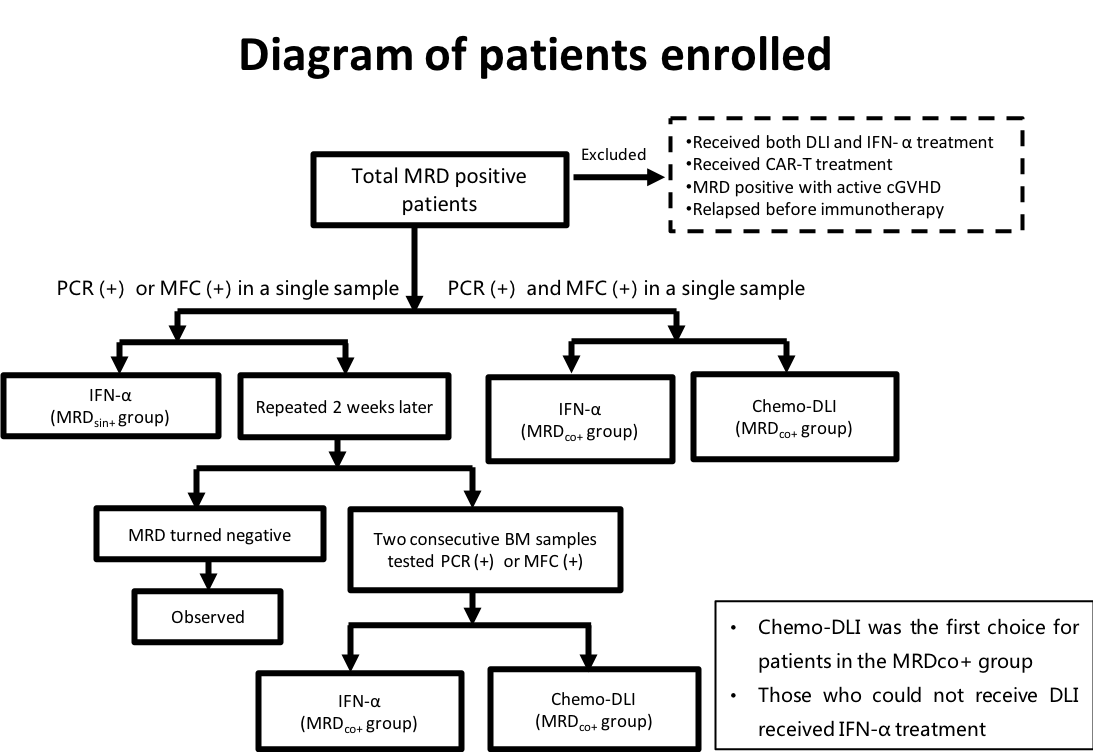


**Methodology**

***MRD monitoring and definition***

MRD was monitored according to leukemia-associated aberrant immune phenotypes (LAIPs) and Wilms’ tumor gene 1 (*WT1*) in patients with acute leukemia. Routine MRD monitoring was performed 1, 2, 3, 4.5, 6, 9, and 12 months post-transplantation and at 6-month intervals thereafter. Because we demonstrated that the combined use of PCR and MFC might achieve higher sensitivity without sacrificing specificity, a patient was considered to have an MRD-positive status when a single BM sample tested positive for MFC or PCR.

***Preemptive IFN-α treatment protocol***

Cases in which a single BM sample tested positive for PCR or MFC were defined as MRD_sin+_ group. Cases in which 2 consecutive BM samples within a 2-week interval tested positive for PCR or MFC or those in which a single BM sample tested positive for both PCR and MFC were defined as MRD_co+_ group.

Patients with MRD_sin+_ were recommended to receive IFN-α treatment. For the patients who did not agree to receive IFN-α treatment, the tests were repeated 2 weeks after positive results for PCR or MFC results were obtained. If 2 consecutive BM samples tested positive for PCR or MFC (MRD_co+_) within a 2-week interval, patients should receive preemptive intervention. In addition, cases in which a single BM sample tests positive for both PCR and MFC (MRD_co+_) should also receive preemptive intervention.

Because the efficacy of chemotherapy plus donor lymphocyte infusion (Chemo-DLI) had been confirmed but the role of IFN-α treatment was still undefined in MRD_co+_ patients when this study started, preemptive Chemo-DLI was the first choice for patients in MRD_co+_ group, and those who could not receive Chemo-DLI because of patient or provider refusal received IFN-α treatment and enrolled in these two studies.

Recombinant human IFN-α-2b injections (Anferon; Tianjin Hualida Biotechnology Co., Ltd., Tianjin, China) were administered subcutaneously for 6 cycles (twice weekly in every 4 weeks cycle) at dosages of 3 million units for patients older than 16 years, and at 3 million units per square meter for those younger than 16 years (capped by 3 million units). Prolonged treatment with IFN-α was permitted at the request of patients. MRD status was monitored 1, 2, 3, 4.5, 6, 9, and 12 months after preemptive IFN-α treatment and at 6-month intervals thereafter.

Adverse events were scored using the National Cancer Institute Common Toxicity Criteria version 4.0, and they were monitored every 1-2 weeks after IFN-α treatment. GVHD was excluded as an adverse event. Study medication with IFN-α was discontinued in any patient with active GVHD (grade II or higher aGVHD or cGVHD with moderate or higher severity), severe infection, grade ≥ 3 toxicity, salvage Chemo-DLI use, relapse, or non-relapse mortality (NRM).

The patients who showed unsatisfactory response to IFN-α treatment can receive salvage Chemo-DLI, if they agreed to receive Chemo-DLI and did not have active GVHD, active infection, and organ failure were eligible for salvage Chemo-DLI: patients had positive MRD again after achieving MRD-negative status or those with persistent and increasing level of MRD after preemptive IFN-α treatment.

**Safety considerations**.

Adverse events were scored using the National Cancer Institute Common Toxicity Criteria version 4.0, and they were monitored every 1-2 weeks after IFN-α treatment. GVHD was excluded as an adverse event. Study medication with IFN-α was discontinued in any patient with active GVHD (grade II or higher aGVHD or cGVHD with moderate or higher severity), severe infection, grade ≥ 3 toxicity, salvage Chemo-DLI use, relapse, or non-relapse mortality (NRM).

**Follow-up:**

Data were censored at the time of relapse, death or the last available follow-up. The expected last follow-up visit was 2 years from the enrollment of the last patients

**Data management and statistical analysis**

The primary endpoint was relapse, and secondary endpoints were NRM, disease-free survival (DFS), and overall survival (OS). This study was planned to detect a relapse rate of 55% in patients with MRD receiving preemptive IFN-α treatment, from the reference rate of 75% in patients with PCR or MFC positivity but did not receive interventions in our previous study, controlling for type I and II error rates at 5% and 10%, respectively. Considering an expulsion rate of 15%, a total of 68 patients was planned to be enrolled.

Comparisons of patient characteristics between the groups were performed using the Mann-Whitney *U*-test for continuous variable and *χ*^2^ and Fisher’s exact tests for categorical data. The probability of survival was calculated using the Kaplan-Meier estimator. The incidences of GVHD were calculated using the cumulative incidence function, with death and relapse as competing risks. Cumulative incidences were estimated for NRM and relapse, to account for competing risks. Relapse was the competing event for NRM and vice versa. Hazard ratios (HRs) for clinical outcomes were estimated from Cox regression analyses. *P* values were 2-sided. The SPSS 24 (SPSS Inc./IBM, Armonk, NY, USA) and the R software package (version 2.6.1; http://www.r-project.org) were used for data analyses.

**Quality assurance**

For studies using eCRFs, investigator will review the data entered by investigational staff for completeness and accuracy. After these actions have been completed and the data has been verified to be complete and accurate, the database will be declared locked and made available for data analysis. Authorization is required prior to making any database changes to locked data.

**Expected outcomes of the study**

Preemptive IFN-α treatment could protect against relapse and improved long-term survival of ALL patients who had MRD after allo-HSCT. Because IFN-α may tend to be started in patients with relatively low leukemia burden, it could not only unlock its therapeutic potential in ALL, but also spare the patients in remission from further therapy. Moreover, IFN-α is a simple treatment with increased accessibility as it could be performed on an outpatient basis.

**Project management**

***Conception/Design****:* Xiao-Dong Mo, and Xiao-Jun Huang

***Patient management****:* Xiao-Dong Mo, Lan-Ping Xu, Xiao-Hui Zhang, Yu Wang, Chen-Hua Yan, Huan Chen, Yu-Hong Chen, Wei Han, Feng-Rong Wang, Jing-Zhi Wang, Kai-Yan Liu, and Xiao-Jun Huang

***Collection and/or assembly of data****:* Xiao-Dong Mo, Si-Ning Liu, and Xue-Yi Luo

***Data analysis and interpretation****:* Xiao-Dong Mo and Si-Ning Liu

**Ethics**

The study was performed in accordance with the Declaration of Helsinki and was approved by the Ethics Committee of Peking University People’s Hospital.

**Informed consent forms**

All patients or the patients’ guardians gave written informed consent before enrollment.
