# Supplementary material for: Preemptive interferon-α treatment could protect against relapse and improve long-term survival of ALL patients after allo-HSCT
Source: Sci Rep. 2020 Nov 19;10:20148. doi: 10.1038/s41598-020-77186-9 (PMC7677364; doi:10.1038/s41598-020-77186-9)
Supplement: Supplementary file 4 — Supplementary Figure 1 Legend. [file 41598_2020_77186_MOESM4_ESM.docx]

**Title:** **Preemptive interferon-α treatment could protect against relapse and improve long-term survival of ALL patients after allo-HSCT**

**Authors:** Sining Liu^1^, Xueyi Luo^1^, Xiaohui Zhang^1^, Lanping Xu^1^, Yu Wang^1^, Chenhua Yan^1^, Huan Chen^1^, Yuhong Chen^1^, Wei Han^1^, Fengrong Wang^1^, Jingzhi Wang^1^, Kaiyan Liu^1^, Xiaojun Huang^1,2^, and Xiaodong Mo (🖂)^1^

**Supplementary figure legend**

**Supplementary figure 1**. Detailed diagram of patients enrolled. Among 35 patients who showed MRD_sin+_ but did not receive IFN-α treatment, immunosuppressions were tapered in 21 patients, but only 1 patient achieved MRD negativity.
