# Supplementary figures and images for: Preemptive interferon-α treatment could protect against relapse and improve long-term survival of ALL patients after allo-HSCT
Source: Sci Rep. 2020 Nov 19;10:20148. doi: 10.1038/s41598-020-77186-9 (PMC7677364; doi:10.1038/s41598-020-77186-9)

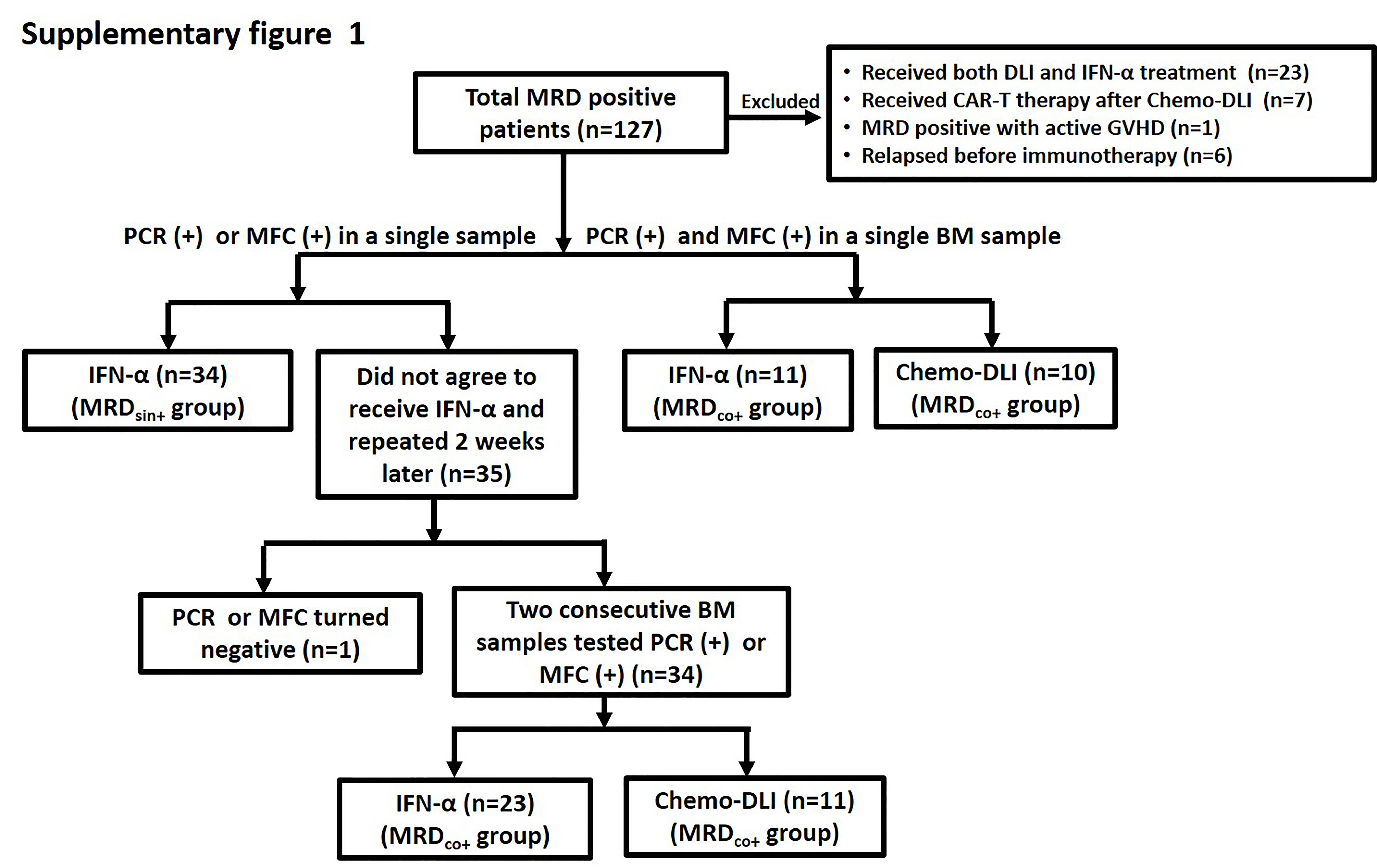

Supplement: Supplementary file 5 — Supplementary Figure 1. [file 41598_2020_77186_MOESM5_ESM.tif]
